# Molecular Characterization of Transcriptional Regulation of *rovA* by PhoP and RovA in *Yersinia pestis*

**DOI:** 10.1371/journal.pone.0025484

**Published:** 2011-09-26

**Authors:** Yiquan Zhang, He Gao, Li Wang, Xiao Xiao, Yafang Tan, Zhaobiao Guo, Dongsheng Zhou, Ruifu Yang

**Affiliations:** 1 State Key Laboratory of Pathogen and Biosecurity, Beijing Institute of Microbiology and Epidemiology, Beijing, People’s Republic of China; 2 State Key Laboratory for Infectious Disease Prevention and Control, National Institute for Communicable Disease Control and Prevention, Chinese Centre for Disease Control and Prevention, Beijing, People’s Republic of China; Tulane University, United States of America

## Abstract

**Background:**

*Yersinia pestis* is the causative agent of plague. The two transcriptional regulators, PhoP and RovA, are required for the virulence of *Y. pestis* through the regulation of various virulence-associated loci. They are the global regulators controlling two distinct large complexes of cellular pathways.

**Methodology/Principal Findings:**

Based on the LacZ fusion, primer extension, gel mobility shift, and DNase I footprinting assays, RovA is shown to recognize both of the two promoters of its gene in *Y. pestis*. The autoregulation of RovA appears to be a conserved mechanism shared by *Y. pestis* and its closely related progenitor, *Y. pseudotuberculosis*. In *Y. pestis*, the PhoP regulator responds to low magnesium signals and then negatively controls only one of the two promoters of *rovA* through PhoP-promoter DNA association.

**Conclusions/Significance:**

RovA is a direct transcriptional activator for its own gene in *Y. pestis*, while PhoP recognizes the promoter region of *rovA* to repress its transcription. The direct regulatory association between PhoP and RovA bridges the PhoP and RovA regulons in *Y. pestis*.

## Introduction

*Yersinia pestis* is one of the most dangerous bacterial pathogens. Humans infected with *Y. pestis* manifest three main forms: pneumonic, septicemic, and bubonic plagues, and it has a very high mortality rate without timely and effective antibiotic treatment [1]. There have been at least three plague pandemics in human history, including the Black Death, which accounted for the death of at least one-third of the European population between 1347 and 1353. Plague remains a great threat to public health because rodent plague epidemics are frequent in various natural plague foci, especially in Asia, America, and Africa, human plague infections are reported every year, and *Y. pestis* can possibly be used as a biowarfare or bioterrorism agent.

PhoP and PhoQ constitute a classic regulatory two-component system [2]. The sensor protein PhoQ responds to low environmental Mg^2+^, acidic pH, and host-secreted antimicrobial peptides, and then phosphorylates the response regulator PhoP. As a transcription factor, phosphorylated PhoP either activates or represses its target genes through binding to their promoter-proximal DNA regions. Intracellular growth of *Y. pestis* in macrophages occurs at early stages of systemic infection [3]. A *phoP* null mutant of *Y. pestis* showed reduced ability to survive in macrophages and human neutrophils, as well as under *in vitro* conditions of low pH, oxidative stress, high osmolarity, and antimicrobial peptides [4], [5], [6]; this mutant is slightly attenuated in mice [4]. As a global regulator, PhoP controls a very complex regulatory cascade in *Y. pestis* [7], [8], [9]. The PhoP regulons in *Y. pestis* and *Salmonella enterica* have considerable differences in terms of the functional changes in PhoP itself, as well as in the architecture of PhoP-dependent promoters. This allows the PhoP regulators to incorporate newly acquired genes into the ancestral regulatory circuits yet retain control of the core regulon members in these two bacteria [8], [9]. The proven direct PhoP targets in *Y. pestis* include several genes that function in detoxification, protection against DNA damage, resistance to antimicrobial peptides, and adaptation to magnesium limitation [7], especially the *mgtCB* and *udg* loci that encodes an Mg^2+^ transport system and a UDP-glucuronate decarboxylase for LPS modification, respectively, required for the replication of *Y. pestis* in macrophages [10]. These PhoP-dependent mechanisms used by *Y. pestis* contribute to intracellular growth of this pathogen.

As a member of the MarR/SlyA family of transcriptional regulators that control the virulence of multiple bacterial pathogens [11], RovA is required for the virulence of all three pathogenic yersiniae (*Y. pestis*, *Y. pseudotuberculosis*, and *Y. enterocolitica*) through regulation of various virulence loci [12], [13], [14], [15], [16]. In *Y. pseudotuberculosis* and *Y. enterocolitica*, RovA stimulates the transcription of *inv*, which encodes an invasin that mediates translocation across the intestinal epithelium [14], [15], [16], [17]. However, the *inv* gene is naturally inactivated in *Y. pestis* due to the insertion of an IS200-like element within its coding region [18]. The *rovA* null mutant of *Y. pestis* is much more attenuated after subcutaneous inoculation than after intranasal or intraperitoneal route, indicating a more important role for RovA in subcutaneous infection than in the pneumonic or systemic one [12]. In *Y. pestis*, RovA stimulates the transcription of the *psaEF*, *psaABC*, and CUS-2 prophage loci [12]. The pH6 antigen encoded by *psaABC* acts as an antiphagocytic factor [19] and plays a more important role in bubonic plague than in the pneumonic and septicemic forms, closely mimicking the role for RovA [12]. The CUS-2 prophage is acquired by the *Y. pestis* ancestor and its genome forms an unstable episome in *Antiqua* and *Medievalis*, and a stably integrated one in *Orientalis* [20], [21]. The acquisition of this prophage does not correlate to flea transmission, but contributes to virulence in mice [20]. The RovA regulator still plays critical roles in the construction and functioning of the bacterial membrane, indicating the regulatory functions of RovA in antibiotic resistance and environmental adaptation [22].

The *rovA* gene transcribes with two distinct promoters, and the autoregulation of *rovA* has been established in *Y. pseudotuberculosis*, but whether both of the two promoters are dependent on RovA is unclear [17], [23]. This study indicates that the autoregulatory mechanism is also conserved in *Y. pestis*, and further discloses that RovA stimulates both of the two promoters. In addition, PhoP responses to low magnesium signals, and recognizes the promoter region of *rovA* to repress its transcription in *Y. pestis*. The direct regulatory association between PhoP and RovA bridges the two distinct complexes of the cellular pathways governed by the two regulators.

## Materials and Methods

### Bacterial strains

The wild-type (WT) *Y. pestis* biovar *Microtus* strain 201, avirulent to humans but highly virulent to mice, was isolated from *Microtus brandti* in Inner Mongolia, China, [24]. The base pairs 41 to 362 of *rovA* (432 bp in total length) or 41 to 631 of *phoP* (672 in total) were replaced with the kanamycin resistance cassette using the one-step inactivation method based on the lambda Red phage recombination system with the helper plasmid pKD46 [25]. This generated the *rovA* and *phoP* mutants of *Y. pestis*, designated as *ΔrovA* and *ΔphoP*, respectively. Chromosomal integration of the mutagenic cassette was confirmed by PCR and sequencing using oligonucleotides external to the integrated cassette. The elimination of pKD46 in the mutants was verified by PCR. All primers used in this study are listed in Table 1.

**Table 1:** Oligonucleotide primers used in this study.

Target	Primers (forward/reverse, 5'–3')
**Construction of mutants**	
*rovA*	TTGGAATCGACATTAGGATCTGATCTAGCACGATTAGTTCAGATTGCAGCATTACACG/CTCAAGCTTATCGATTAGGCCTGATAACACTGCAATTTCATGTAACGCACTGAGAAGC
*phoP*	ATGCGGGTTCTGGTTGTGGAAGATAACGCGTTGTTGCGTCAGTTGTGTCTCAAAATCTCTG/CTAGTTGACGTCAAAACGATATCCCTGACCACGAATAGTCGAAAGCCGCCGTCCCGTCAAG
**Complementation of mutants**	
*phoP*	GCGGGATCCCGTGAACATCGCCTATCGTC/GCGAAGCTTTGCCACTGTGCCAGACTG
*rovA*	GATCGATATCGCTCAGTTGCCGCCTTC/GATCGGATCCCTGCTGTGAATAAAGTCTTTGAAC
**RT-PCR**	
*phoP*	TTGTTGCGTCACCATCTG/GGCTTAACCCGTCTTCAC
*rovA*	TTACCACCAGAGCAATCACAG/ATCACGCCATCAACCTGTTC
**LacZ fusion**	
*rovA*	GCGGGATCCCGTTCGTTACTCTGCCCATC/GCGAAGCTTTTGTGATTGCTCTGGTGGTAAAC
**Primer extension**	
*rovA*	/GTATCCTCATTACCCAGCATCG
	/GTGCTAGATCAGATCCTAATGTCG
**Protein expression**	
*rovA*	GCGGGATCCTTGGAATCGACATTAGGATC/GCGGTCGACTTACTTAGTTTGTAATTGAATA
*phoP*	GCGGGATCCATGCGGGTTCTGGTTGTGG/GCGAAGCTTTTAGTTGACGTCAAAACGATATCCC
**EMSA**	
*rovA*	CGTTCGTTACTCTGCCCATC/TGTGATTGCTCTGGTGGTAAAC
	TGCTCCCGACGCTAAGTG/TAGAAAATTTGTTCCCCTCGAC
	CTGAAAGCGAGGCGATGC/TCAGCCGATGGTCAATTAATGC
**DNase I footprinting**	
*rovA*	TGCTCCCGACGCTAAGTG/TAGAAAATTTGTTCCCCTCGAC
	CTGAAAGCGAGGCGATGC/TCAGCCGATGGTCAATTAATGC
	ACCAAATCTGAAAGCGAGGCG/GTGCTAGATCAGATCCTAATGTCG

A PCR-generated DNA fragment containing the *rovA* or *phoP* coding region with its promoter-proximal region (∼500 bp upstream the coding sequence) and transcriptional terminator (∼300 bp downstream) were cloned into the pACYC184 vector that harbors a chloramphenicol resistance gene (GenBank accession number X06403), as verified by DNA sequencing. The recombinant plasmid was subsequently introduced into *ΔrovA* and *ΔphoP*, yielding the complemented mutant strains *C-rovA* and *C-phoP*, respectively.

### Bacterial growth

The original chemically defined TMH medium [26] [called “high magnesium, neutral pH” (I)] and its different modifications were used for *Y. pestis* cultivation. The 20 mM MgCl_2_ in the original TMH was changed to 10 µM to simulate the ‘low magnesium’ (II) condition. To simulate ‘mild acidic pH’ (III), the pH value of 7.2 in the original TMH was changed to 5.8. Overnight cell culture with an optical density (OD_620_) of about 1.0 in each medium was diluted 1∶20 into 18 ml of the corresponding fresh medium for further cultivation.

**Figure 1:**
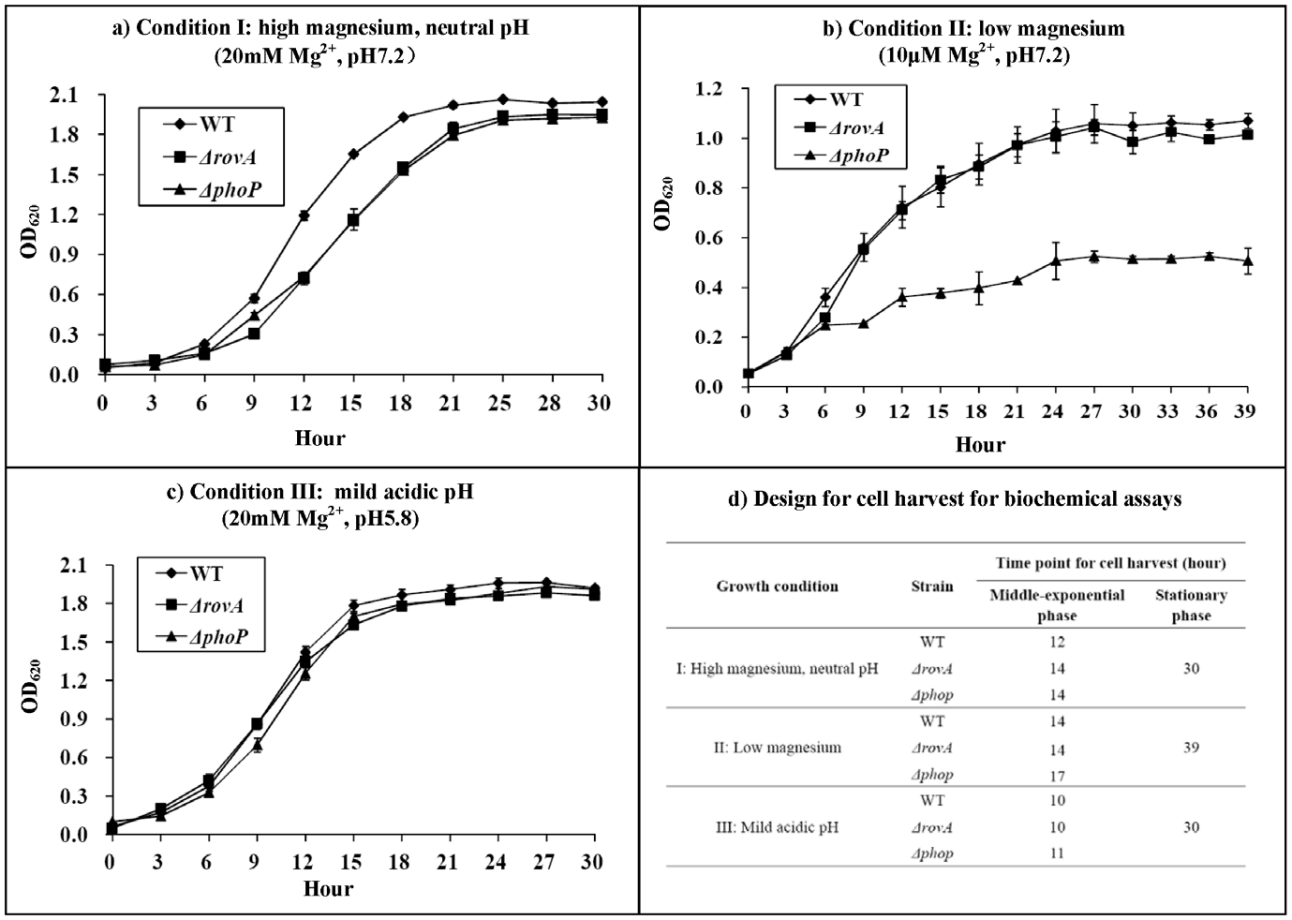
Bacterial growth curves at 26°C. Overnight cell culture with an OD_620_ value of about 1.0 in each medium was diluted 1∶20 into 18 ml of the corresponding fresh medium. Bacteria were then grown at 26°C with shaking at 230 rpm, and the OD_620_ values were monitored for each culture with a 3 or 4 h interval until the cultures reached the stationary growth phase (a, b, and c). Experiments were done with three biological replicates. Shown also is the design (d) for cell harvest for subsequent biochemical assays.

Cells were harvested at the middle-exponential or stationary phase for the followed primer extension or LacZ fusion assay. For cell harvest at the middle-exponential phase, bacteria were grown at 26°C with shaking at 230 rpm to enter the exponential phase; and then, half of the cell cultures were incubated at 37°C for 3 h and the remaining half were allowed to grow continuously at 26°C for 3 h. For harvest at the stationary phase, bacteria were grown at 26°C to enter the stationary phase; the cell cultures were then divided to grow at 37 and 26°C, respectively, for 3 h as above. The detailed time points for cell harvest were defined according to the bacterial growth curves (Fig. 1). The above cultures grown at 37 and 26°C were designated as “shift from 26 to 37°C (#)” and “26°C continuously (&),” respectively, so as to determine the effect of temperature on gene transcription.

### Primer extension assay

Total bacterial RNAs were extracted using the TRIzol Reagent (Invitrogen) [7], [25]. Immediately before harvesting, bacterial cultures were mixed with RNAprotect Bacteria Reagent (Qiagen) to minimize RNA degradation. RNA quality was monitored by agarose gel electrophoresis and RNA quantity was determined by spectrophotometry. For the primer extension assay [7], [25], an oligonucleotide primer complementary to a portion of the RNA transcript of *rovA* gene was employed to synthesize cDNAs from the RNA templates. About 10 µg of the total RNA from each strain was annealed with 1 pmol of [γ−^32^P] end-labeled reverse primer using a Primer Extension System (Promega) according to the manufacturer’s instructions. The same labeled primer was also used for sequencing with the fmol® DNA Cycle Sequencing System (Promega). The primer extension products and sequencing materials were concentrated and analyzed in a 6% polyacrylamide/8 M urea gel. The result was detected by autoradiography (Kodak film).

### LacZ fusion and β-galactosidase assay

The 889 bp promoter-proximal DNA region of *rovA* was obtained by PCR with the ExTaq™ DNA polymerase (Takara) using *Y. pestis* 201 genome DNA as the template. PCR fragments were then directionally cloned into the *Eco*RI and *Bam*HI sites of low-copy-number plasmid pRW50 that harbor a tetracycline resistance gene and a promoterless *lacZ* reporter gene [27]. Correct cloning was verified by DNA sequencing. An empty pRW50 plasmid was also introduced into each strain tested as the negative control. The *Y. pestis* strains transformed with the recombinant plasmids and the empty pRW50 plasmid were grown as previously described to measure the β-galactosidase activity in the cellular extracts using the β-Galactosidase Enzyme Assay System (Promega) [25]. Assays were performed with at least three biological replicates.

### Purification of PhoP and RovA proteins

Preparation of the purified PhoP and RovA proteins were performed as previously described [7], [25]. The entire coding region of the *phoP* and *rovA* genes of strain 201 was directionally cloned into the *BamH*I and *Hind*III sites of plasmid pET28a (Novagen). The recombinant plasmid encoding the 6× His-tagged PhoP and RovA proteins (His-PhoP and His-RovA, respectively) were transformed into *Escherichia coli* BL21λDE3 cells. Expression of His-PhoP or His-RovA was induced by the addition of 1 mM IPTG (isopropyl-b-D-thiogalactoside). The overproduced proteins were purified under native conditions using an Ni-NTA Agarose Column (Qiagen). The purified protein was concentrated with the Amicon Ultra-15 centrifugal filter device (Millipore) and the protein purity was verified by SDS-PAGE.

### Gel mobility shift assay (EMSA)

The *rovA* promoter-proximal regions were amplified by PCR. For EMSA [7], [25], the 5' ends of DNA were labeled using [γ−^32^P] ATP and T4 polynucleotide kinase. DNA binding was performed in a 10 µl reaction volume containing binding buffer [1 mM MgCl_2_, 0.5 mM EDTA, 0.5 mM DTT, 50 mM NaCl, 10 mM Tris-HCl (pH 7.5) and 0.05 mg/ml poly-(dI-dC)], labeled DNA (1000 to 2000 c.p.m/µl), and increasing amounts of the His-PhoP or His-RovA protein. Three controls were included in each EMSA experiment: 1) cold probe as specific DNA competitor (the same promoter-proximal DNA region unlabeled), 2) negative probe as nonspecific DNA competitor (the unlabeled coding region of the 16S rRNA gene), and 3) nonspecific protein competitor [rabbit anti-F1-protein polyclonal antibodies]. After incubation at room temperature for 30 min, the products were loaded onto a native 4% (w/v) polyacrylamide gel and electrophoresed in 0.5× TBE buffer for about 50 min at 220 V. Radioactive species were detected by autoradiography after exposure to Kodak film at −70°C.

### DNase I footprinting

For DNase I footprinting [7], [25], the *rovA* promoter-proximal DNA regions with a single ^32^P-labeled end were PCR amplified with either the sense or antisense primer being end-labeled. The PCR products were purified using MinElute reaction cleanup columns (Qiagen). Increasing amounts of His-PhoP or His-RovA were incubated with the purified, labeled DNA fragment (2 to 5 pmol) for 30 min at room temperature, in a final 10 µl reaction volume containing the binding buffer used in EMSA. Before DNA digestion, 10 µl of Ca^2+^/Mg^2+^ solution (5 mM CaCl_2_ and 10 mM MgCl_2_) was added, followed by incubation for 1 min at room temperature. The optimized RQ1 RNase-Free DNase I (Promega) was then added to the reaction mixture, and the mixture was incubated at room temperature for 40 to 90 s. The reaction was quenched by adding 9 µl of stop solution (200 mM NaCl, 30 mM EDTA, and 1% SDS), followed by incubation for 1 min at room temperature. The partially digested DNA samples were extracted with phenol/chloroform, precipitated with ethanol, and analyzed in 6% polyacrylamide/8 M urea gel. Protected regions were identified by comparison with the sequence ladders. For sequencing, we used the *fmol*® DNA Cycle Sequencing System (Promega). The templates for sequencing were the same as the DNA fragments of DNase I footprinting assays. Radioactive species were detected as previously described.

## Results

### Mutation and complementation

Real-time RT-PCR experiments were performed to assess the relative mRNA levels of *phoP* and *rovA* in the corresponding WT, mutant, and complemented mutant strains. The *phoP* transcript was lacking in *ΔphoP*, but was restored in *C-phoP* relative to WT, and moreover similar results were observed for the *rovA* transcript in WT, *ΔrovA*, and *C-rovA* (data not shown). These data indicates the successful mutation and complementation of *phoP* and *rovA*.

To test whether the *phoP* or *rovA* mutation had the polar effect, the primer extension assays were conducted to detect the yield of the *rovA* primer extension product that represents the *rovA* transcriptional levels in the corresponding WT, mutant, and complemented mutant strains (Figure S1). As determined by several distinct methods (see below), the *rovA* gene was positively regulated by RovA under condition I, whereas it was under the negative control of PhoP under condition II. As determined by the primer extension assays herein, the *rovA* transcription under condition I was significantly repressed in *ΔrovA* relative to WT, and restored in *C-rovA*; its transcription under condition II was significantly elevated in *ΔphoP* relative to both *C-phoP* and WT (Figure S1). The *rovA* gene yielded almost the same transcriptional levels between the paired WT/*ΔphoP* or WT/*ΔrovA* strains. This complementation analysis confirmed that the observed PhoP or RovA-dependent transcription of *rovA* was due to the *phoP* or *rovA* mutation, respectively, rather than a polar mutation.

### Growth of WT, ΔrovA, and ΔphoP

The growth curves of the WT, *ΔrovA*, and *ΔphoP* strains grown at 26°C under three different conditions I, II, and III were determined (Fig. 1). Under condition I, both *ΔrovA* and *ΔphoP* exhibited growth rates lower than WT (Fig. 1a). Under condition II, growth restriction was observed for *ΔphoP* rather than *ΔrovA* relative to WT (Fig. 1b). The three strains showed indistinguishable growth rates under condition III (Fig. 1c). For each strain, bacterial growth was impeded under suboptimal conditions II and III relative to the original condition I. In particular, bacterial cells exhibited very poor growth when each of strain was grown under condition II.

Bacterial cells were harvested at the middle-exponential or stationary phase for the following cell culture-related biochemical assays, and the time points for cell harvest were defined strictly according to the growth curves (Fig. 1d). It should be noted that bacterial cells grown under different conditions or those of different isogenic stains were harvested at the identical growth phase, rather than the identical optical density, which would devoid the secondary effects of growth rate or phase. In addition, temperature upshift from 26 to 37°C was designed prior to cell harvest, generating 26 (“26°C continuously”) and 37°C (“shift from 26 to 37°C”) grown cells.

### Transcription of rovA under different temperatures

**Figure 2:**
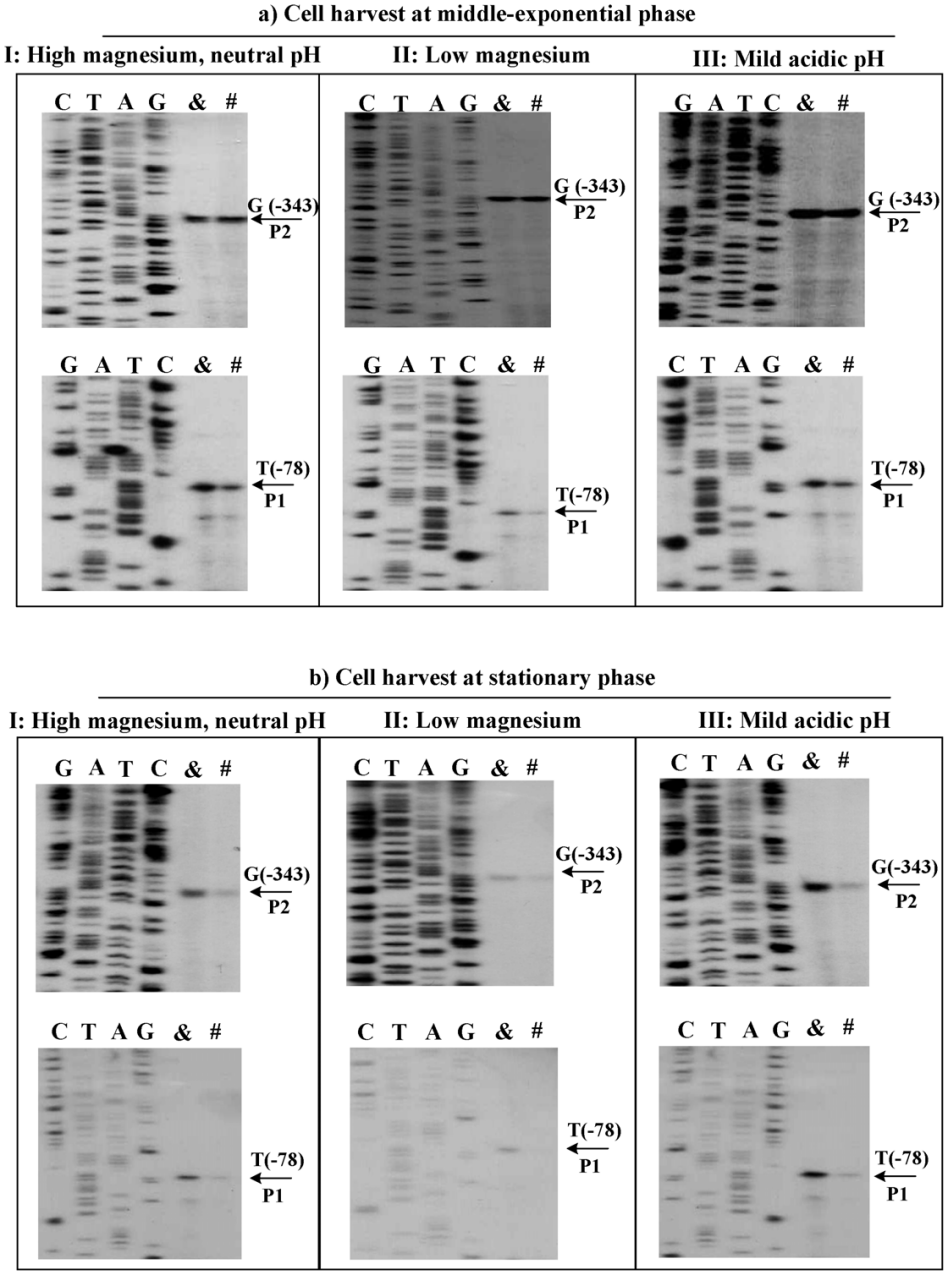
Transcription of *rovA* upon temperature shift from 26 to 37°C. Two oligonucleotide primers were designed to be complementary to the RNA transcript of *rovA*. The primer extension products were analyzed with 8 M urea−6% acrylamide sequencing gel. Lanes C, T, A, and G represent the Sanger sequencing reactions. Only the WT strain was tested to grow under conditions I, II and III, respectively. Bacterial cells were harvested at the middle-exponential (a) or stationary (b) phase. Temperature upshift was designed prior to cell harvest, generating two kinds of cultures: ‘26°C continuously’ (&) and ‘shift from 26 to 37°C’ ( #). Detected were the two promoters P1 and P2 located at 78 (nucleotide T ) and 343 (G) bp upstream of *rovA*, respectively. Images shown are representative of the results from at least three biological replicates.

The primer extension experiments (Fig. 2) were then conducted to determine the yields of primer extension product of *rovA* (i.e., the relative *rovA* transcription levels, or the relative *rovA* promoter activities) in WT upon the above temperature upshift. The primer extension assay detected two transcriptional start sites located at 343 and 78 bp upstream of *rovA* (Fig. 2); therefore, two promoters (named P2 and P1, respectively) were transcribed for *rovA*. At the middle-exponential growth phase (Fig. 2a), the P2 promoter activity showed no obvious change upon the temperature upshift under conditions I, II, and III; yet, the P1 promoter activity decreased upon temperature upshift under all the three growth conditions. At the stationary growth phase (Fig. 2b), the promoter activities of both P2 and P1 decreased upon temperature upshift under all the three growth conditions. In conclusion, the temperature shift from 26 to 37°C triggered the down-regulation of both P2 and P1 promoters of *rovA* at the stationary growth phase. However, the down-regulatory effect occurred only for P2 at the middle-exponential growth phase.

For subsequent experiments, only bacterial cultures of “shift from 26 to 37°C” were analyzed, as 37°C is the temperature during human infections.

### Autoregulation of RovA

**Figure 3:**
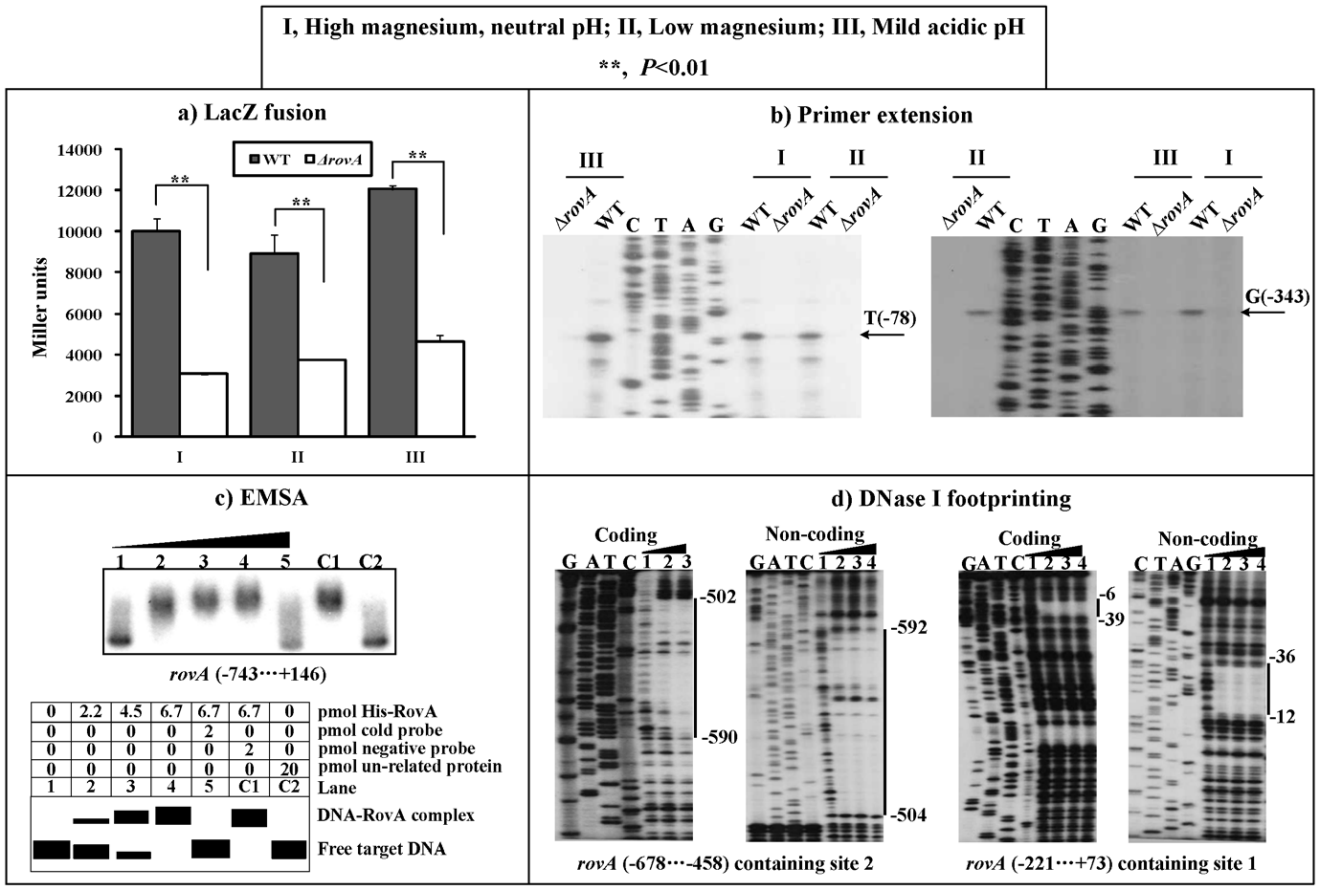
RovA stimulated transcription of its own gene. Only the bacterial cells of “shift from 26 to 37°C” at the middle-exponential growth phase were analyzed herein. **a) LacZ fusion.** A promoter-proximal region 743 bp upstream to 146 bp downstream of *rovA* was cloned into pRW50 containing a promoterless *lacZ* reporter gene, and then transformed into WT or *ΔrovA* to determine the β-galactosidase activity in cellular extracts. Shown are the *rovA* promoter activities (Miller units) in *ΔrovA* or WT grown under conditions I, II and III, respectively. **b) Primer extension.** The P1 and P2 promoters of *rovA* were detected in *ΔrovA* or WT grown as described above. Lanes C, T, A, and G represent the Sanger sequencing reactions. **c) EMSA.** The radioactively labeled DNA fragment from the 743^rd^ bp upstream to the 146^th^ bp downstream of *rovA* was incubated with increasing amounts of purified His-RovA protein, and then subjected to 4% (w/v) polyacrylamide gel electrophoresis. The band of free DNA disappeared with increasing amounts of His-RovA protein, and a retarded DNA band with decreased mobility turned up, which presumably represented the DNA-RovA complex. Shown on the lower side of the figure is the schematic representation of the EMSA design. **d) DNase I footprinting.** Labeled coding or non-coding DNA probes were incubated with increasing amounts of purified His-RovA (Lanes 1, 2, 3, and 4 containing 0, 4, 8, and 12 pmol, respectively), and subjected to DNase I footprinting assay. Lanes G, A, T, and C represent the Sanger sequencing reactions. The protected regions (vertical bars) are indicated on the right side of the image. The negative numbers indicate the nucleotide positions upstream of *rovA*.

A *rovA-lacZ* fusion vector, containing the 889 bp promoter-proximal region of *rovA* and the promoterless *lacZ*, was transformed into both WT and *ΔrovA* to compare the *rovA* promoter activities in these two strains grown under conditions I, II, and III, respectively (Fig. 3a). Under all the three conditions, the expression of *rovA* significantly decreased in *ΔrovA* relative to WT. In addition, the primer extension experiments (Fig. 3b) were conducted to compare the yields of primer extension product of *rovA* in WT and *ΔrovA*; the activities of both P1 and P2 promoters were under the positive control of RovA under all the three growth conditions, which was consistent with the above *lacZ* fusion data. Interestingly, the *rovA* transcription in WT were up-regulated under condition III relative to the other two conditions (Fig. 2a and 2b), and thus mild acid appeared to stimulate the *rovA* expression.

The 889 bp promoter-proximal region of *rovA* tested in the LacZ fusion assay was amplified, radioactively labeled, and subjected to EMSA with a purified His-RovA protein (Fig. 3c). The results show that His-RovA is able to bind to this DNA fragment in a dose-dependent manner *in vitro* (Fig. 3c). As further determined by DNase I footprinting (Fig. 3d), the purified His-RovA protected two distinct regions upstream of *rovA* against DNase I digestion in a dose-dependent manner. These two footprints, located from 592 to 502 bp (RovA site 2) and from 39 to 6 bp (Site 1) upstream of *rovA*, respectively, were considered RovA sites. Taken together, RovA is able to recognize all the promoters of its own gene to stimulate their activity in *Y. pestis*.

**Figure 4:**
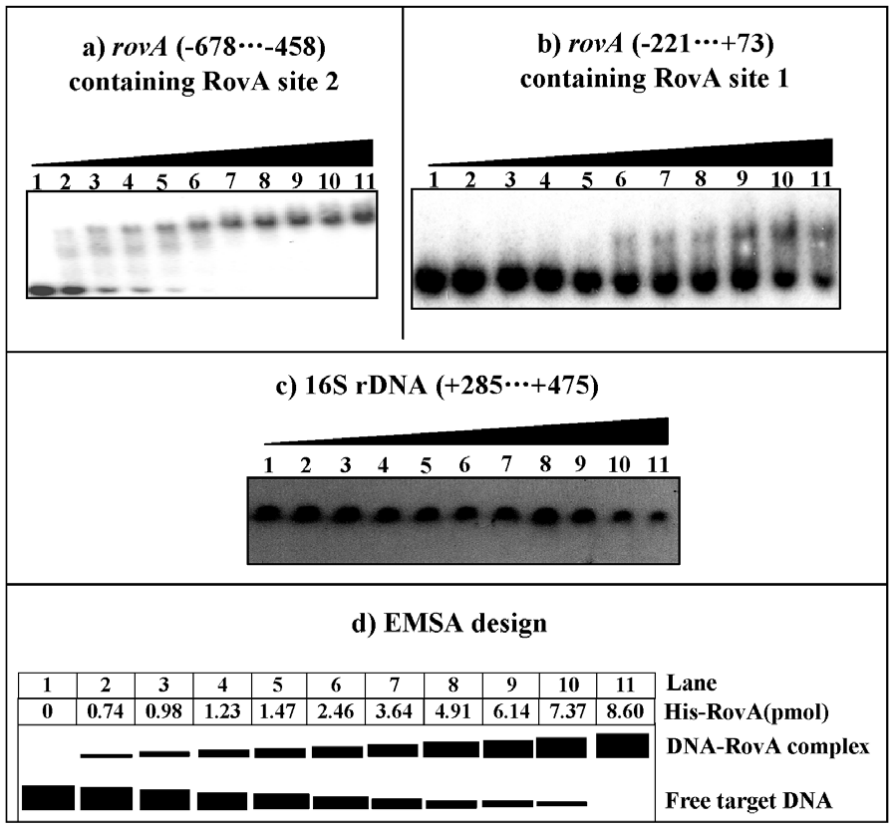
Affinity of RovA to its Sites 1 and 2. Labeled DNA fragments (a and b), which contained RovA Sites 1 and 2 respectively, were incubated with increasing amounts of purified His-RovA. The EMSA experiment was conducted with a coding region of the 16S rRNA gene (c) as the negative control. Also shown is the schematic representation of the EMSA design (d).

To test the affinity of RovA to Sites 1 and 2, EMSA was performed with two distinct *rovA* upstream DNA fragments containing Sites 1 and 2, respectively (Fig. 4). DNA retardation occurred at 0.74 pmol of His-RovA for the fragment containing Site 2 (Fig. 4a), whereas it was observed at 2.46 pmol for that containing Site 1 (Fig. 4b). This indicated that RovA had a much higher affinity to Site 2 than Site 1. The RovA proteins at all amounts used could not bind to the 16S rDNA fragment as the negative control, confirming the specificity of EMSA in this study (Fig. 4c).

### Negative regulation of rovA by PhoP

**Figure 5:**
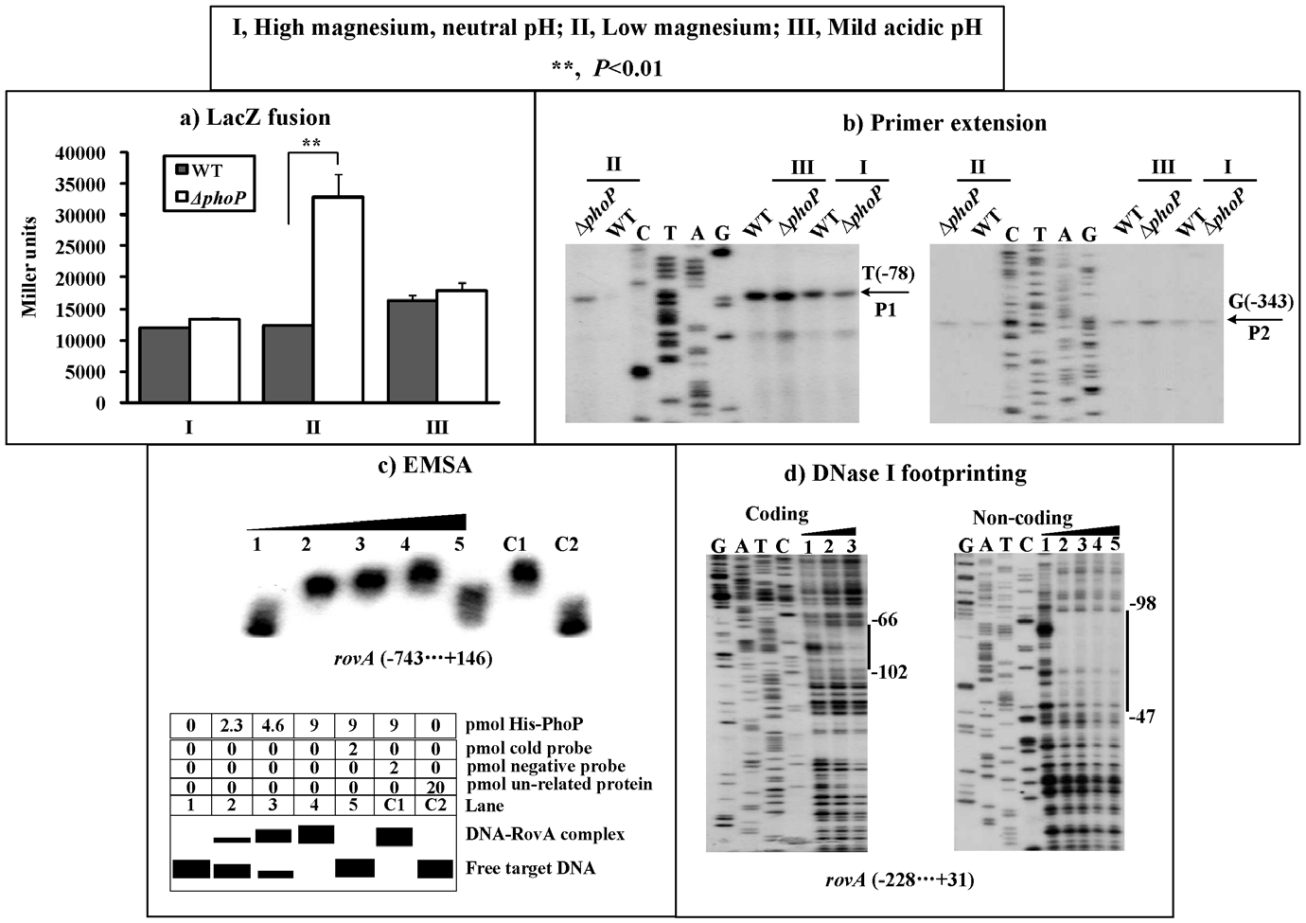
PhoP repressed *rovA* transcription. Only the bacterial cells of “shift from 26 to 37°C” at the middle-exponential growth phase were analyzed herein. **a) LacZ fusion**. The *rovA-lacZ* fusion vector as described in Fig. 3a was transformed into WT or *ΔphoP* to determine the *rovA* promoter activity (Miller units) when the bacteria were grown under conditions I, II and III, respectively. **b) Primer extension.** The P1 and P2 promoters of *rovA* were detected in *ΔphoP* or WT grown as described above. Lanes C, T, A, and G represent the Sanger sequencing reactions. **c) EMSA.** The labeled promoter-proximal fragment of *rovA* as described in Fig. 3c was incubated with increasing amounts of purified His-PhoP. Shown on the lower side is the schematic representation of the EMSA design. Lanes C, T, A, and G represent the Sanger sequencing reactions. **d) DNase I footprinting.** The labeled DNA fragment from 228 bp upstream to 31 bp downstream of *rovA* was incubated with increasing amounts of His-PhoP. Lanes 1, 2, 3, 4 and 5 contained 0, 34.6, 41.5, 48.4 and 59.3 pmol, respectively. Vertical bars indicate the protected regions, whereas the negative numbers denote the nucleotide positions upstream of *rovA*. Lanes C, T, A, and G represent the Sanger sequencing reactions.

The *rovA*-lacZ fusion vector (Fig. 3a) was transformed into both WT and *ΔphoP* to compare the *rovA* promoter activities in the two strains grown under conditions I, II, and III (Fig. 5a). Under conditions I and III, there was no significant difference in the *rovA* promoter activities in the WT and *ΔphoP* strains. Under condition II, the expression of *rovA* was significantly enhanced in the *ΔphoP* relative to the WT. Further primer extension experiments for *rovA* (Fig. 5b) again detected the two promoters, P1 and P2, when the bacterial cells were grown under the three conditions. P1 activity was under the negative control of PhoP under condition II, and independent of this regulator under the other two growth conditions. PhoP had no effect on P2 activity under all the three conditions. The EMSA assay disclosed that the purified His-PhoP protein was able to bind to the 889 promoter-proximal region of *rovA* in a dose-dependent manner *in vitro* (Fig. 5c). Subsequent DNase I footprinting experiments (Fig. 5d) indicated that His-PhoP protected a single region located from 102 to 47 bp upstream of *rovA*. This footprint was considered the PhoP site. Therefore, the *rovA* transcription is negatively controlled by the PhoP regulator under the low magnesium conditions through the PhoP-promoter DNA association.

### Promoter structure of rovA

**Figure 6:**
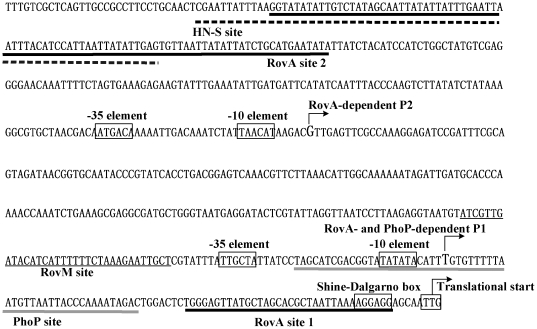
Organization of the *rovA* promoter-proximal region. The DNA sequence was derived from the genomic data of *Y. pestis* 91001 and the start codon was shown at the 3' terminal. The bent arrows indicate the two promoters P1 and P2 (transcription start sites). Predicted promoters −10 and −35 elements, and Shine-Dalgarno box are enclosed in boxes. The RovA, PhoP, H-NS, and RovM sites are underlined with different lines. The H-NS [17], [23] and RovM [36] sites are derived from those determined in *Y. pseudotuberculosis*, since the *Y. pestis rovA* promoter-proximal region is essentially identical to the *Y. pseudotuberculosis* one.

In this study, DNase I footprinting experiments precisely determined the PhoP and RovA sites for *rovA*. The primer extension assays mapped two promoters (P1 and P2) for *rovA*. Accordingly, the core promoter −10 and −35 elements for RNA polymerase recognition were predicted. Collection of data on the translation/transcription start sites, Shine-Dalgarno sequence (a ribosomal binding site in the mRNA), promoter −10 and −35 elements, as well as PhoP and RovA sites enabled us to depict the organization of PhoP and RovA-dependent promoters of *rovA* characterized herein (Fig. 6). The *Y. pestis rovA* promoter-proximal region is essentially identical to the *Y. pseudotuberculosis* one [28]. The two RovA sites within the *rovA* promoter-proximal region in *Y. pestis* are very similar to, although slightly different from, those determined in *Y. pseudotuberculosis* [17]. *Y. pseudotuberculosis* and *Y. pestis* have the same P2 promoter, but P1 in *Y. pestis* is 2 bp upstream of that in *Y. pseudotuberculosis*. The slight differences observed in the P1 sites and the RovA site sequences might be due to the personal inclination during DNA sequence reading.

## Discussion

### Bacterial growth under magnesium-limitation conditions

The magnesium cation (Mg^2+^) is one of the essential elements for bacterial cell growth due to its function as a cofactor of enzymes. When grown under the condition II, *Y. pestis* cells exhibited poor growth, and moreover a extremely heavy restriction of growth was observed for *ΔphoP*. The Mg^2+^ transport systems are positively controlled by the Mg^2+^-responsive PhoP regulator in *Y. pestis* [7], [29], and the *phoP* mutation will impair the magnesium homeostasis of *ΔphoP* under Mg^2+^-limiting environments [2].

### Regulation of rovA by growth temperature

The three pathogenic yersiniae *Y. pestis, Y. pseudotuberculosis*, and *Y. enterocolitica* are ranked at different linkages in the evolution of the *Yersinia* genus [30]. The *Y. pseudotuberculosis*–*Y. pestis* clade diverged from *Y. enterocolitica* hundreds of millions of years ago, whereas *Y. pestis* from *Y. pseudotuberculosis* within thousands of years [31], [32]. Consistently, *Y. pseudotuberculosis* and *Y. pestis* are very divergent from *Y. enterocolitica*, but share a very high level of genomic homology with each other.

As previously shown [17], [33], two (P1 and P2) and three promoters are transcribed for *rovA* in *Y. pseudotuberculosis* and *Y. enterocolitica*, respectively, at room temperature (20 to 26°C), but a down-regulation was observed at 37°C. In addition to the mechanism of transcriptional regulation, the temperature control of *rovA* expression occur also at the post-transcriptional level in *Y. pseudotuberculosis* [34]. In this study, P1 and P2 were also detected for *rovA* in *Y. pestis.* The temperature shift from 26 to 37°C triggered the down-regulation of P1 promoter in *Y. pesti*s at both stationary and middle-exponential growth phase. However, the down-regulation of P2 upon temperature upshift occurred only at the stationary phase rather than the middle-exponential one, indicating a growth phase-dependent effect for P2.

### Regulation of rovA by transcriptional regulators

This study confirms that the autoregulation of *rovA* in *Y. pestis* is identical to that reported in *Y. pseudotuberculosis*, and further discloses that RovA stimulates the activity of both of the two promoters of its own gene in *Y. pestis*.

The nucleoid-associated protein H-NS silences target genes by selectively targeting their upstream DNA sequences with GC contents lower than that of the resident genome [35]. Similarly, *Y. pseudotuberculosis* H-NS binds to a long DNA region upstream of P2 (Fig. 6), and represses the *rovA* transcription [17], [23]. In addition, a LysR-type regulator RovM specifically binds to a short region closely upstream of the −35 element of P1 and far downstream of P2 (Fig. 6), and participates in the repression of *rovA* in *Y. pseudotuberculosis* [36]. Interestingly, the cooperation of RovM and H-NS is required for efficient silencing of *rovA* transcription [36]. It seems that the interaction of RovM and H-NS on the *rovA* promoter-proximal regions, which is accompanied by H-NS/RovM-DNA association, promotes the formation of a stable repressor complex to silence the *rovA* transcription [36].

The RovA Site 2 overlaps the H-NS site for *rovA* (Fig. 6), and RovA alleviates the H-NS-mediated repression of *rovA* by antagonizing the H-NS-promoter DNA association [17], [23]. The RovA Site 2 is upstream of the −35 elements of both P1 and P2, and thus the transcriptional activation of P1 and P2 by RovA is a Class I stimulation dependent on the RNA polymerase α subunit C-terminal domain (αCTD) for function [23], [37]. With the development of RovA autostimulation, the cellular RovA reaches a certain level; in this case, the low-affinity Site 1 is occupied by RovA. Notably, Site 1 is downstream of both P1 and P2, and accordingly the RovA Site 1 association blocks the entry of the RNA polymerase, which destroys the “endless” RovA-mediated activation of *rovA* transcription [23], [37], [38]. This RovA concentration-dependent regulation of its own gene allows the bacterium to finely modulate the cellular RovA levels for the most favorable production of RovA-dependent virulence factors [23], [37], [38].

As shown in this study, the PhoP regulator recognizes a single site within the *rovA* promoter-proximal region, and negatively controls the *rovA* transcription under magnesium-limiting conditions. It is further confirmed that PhoP as the responsive regulator of the PhoP/PhoQ two-component system responds to low magnesium signals [7], [8], [9] rather than magnesium-rich or acidic pH conditions. An 18-bp PhoP box sequence (TGTTTAWN_4_ TGTTTAW), which is consisted of a direct repeat of the hepta-nucleotide consensus (underlined), has been established previously in *Y. pestis* [7]. This box consensus represents the conserved signals for PhoP recognition in *Y. pestis*. Herein, a PhoP box-like sequence (TGTGTTT TTAATGTTAAT ) is found in the PhoP site for *rovA*. Notably, the promoter activity of P1, but not P2, is dependent on PhoP. The PhoP site overlaps the −10 region of the P1 promoter, and thus the PhoP-promoter association is thought to block RNA polymerase-DNA association, thereby repressing the transcription of *rovA*. This mode of regulator-promoter DNA interaction for transcriptional repression is frequently observed in transcriptional repressors, such as Fur [39] and Zur [40] in *Y. pestis*. PhoP and RovA control distinct complexes of cellular pathways, especially including those involved in virulence and host-adaptation [7], [8], [9], [12], [17], [41]. The two regulons governed by PhoP and RovA, respectively, have evolved to merge into a single global regulatory circuit, due to the direct transcriptional association between PhoP and RovA.

The *rovA* upstream DNA regions are identical in *Y. pestis* and *Y. pseudotuberculosis*, and moreover all the four regulators (RovA, PhoP, H-NS, and RovM) involved in the regulation of *rovA* are extremely conserved in these two bacteria. Therefore, the mechanisms that regulate *rovA* discussed above are conserved in these two bacteria.

*S. typhimurium* has the homolgous gene (named *slyA*) of *rovA*. SlyA and PhoP formed a complex positive feedback circuit in *S. typhimurium* [42]. The *slyA* transcription is activated by the PhoP/PhoQ system under low Mg^2+^ conditions [43], [44], and PhoP footprints the *slyA* upstream region [43], which indicating that PhoP stimulates *slyA* directly through PhoP-promoter DNA association. H-NS binds to the *phoP* upstream region to silence the transcription of *phoPQ* operon under high Mg^2+^ conditions [45]. PhoP binds to the *phoP* upstream region and activates (i.e., autoregulates) the *phoP* transcription under low Mg^2+^ conditions [46], [47]. SlyA also footrpints with the *phoP* upstream region, and competes with H-NS since they share the same footprint that is adjacent to the PhoP site within the *phoP* upstream region [42]. Thus, the association between the *phoP* upstream region and SlyA will facilitate PhoP binding to the PhoP site by reducing the inhibitory activity of the H-NS protein [42]. Whether *Y. pestis* employs a regulatory feedback circuit involving in RovA and PhoP/PhoQ needs to be elucidated. In addition to *Y. pestis* and *Y. pseudotuberculosis,* the genus *Yersinia* still contains another pathogenic species, i.e., *Y. enterocolitica*, which shows a widely genetic diversity from the *Y. pseudotuberculosis*/*Y. pestis* clade. The DNA upstream of *rovA* in *Y. enterocolitica* differs greatly from the relevant *Y. pseudotuberculosis*/*Y. pestis* DNA. The *Y. enterocolitica rovA* gene is transcribed with the P1 and P2 promoters that are identical to those in *Y. pseudotuberculosis*/*Y. pestis*, and moreover a third promoter P3 downstream of P1 is detected. The differences in the promoters result in significantly lower levels of *rovA* transcription in *Y. enterocolitica*. H-NS binds to two regions upstream of *rovA* to repress the *rovA* transcription in *Y. enterocolitica*. H-NS shows much lower affinities for either of the two sites than for the reported single *Y. pseudotuberculosis*/*Y. pestis* site. RovA stimulates the *rovA* transcription in *Y. enterocolitica* although the lack of observable RovA binding to the *Y. enterocolitica* promoter. RovM binds to a single region upsteam of *rovA* to repress the *rovA* transcription, as reported for *Y. pseudotuberculosis*. Together, the *cis*-acting DNA region of *rovA* has undergone great genetic variation between *Y. enterocolitica* and *Y. pseudotuberculosis*/*Y. pestis*, which will lead to the remodeling in the mechanisms for controlling the *rovA* transcription, although the relevant trans-acting factors (H-NS, RovA, and RovM) are highly conserved in the three pathogenic yersiniae.

## Supporting Information

Figure S1 **Primer extension assay for validation of non-polar mutation.** The *rovA* or *phop* null mutant (*ΔrovA* or *Δphop*, respectively) was generated from the wild-type strain 201 (WT), and then the corresponding complemented mutant strain (*C-rovA* or *C-phop*, respectively) was constructed. As determined by several distinct methods (see the text of manuscript), the P1 promoter of *rovA* was positively regulated by RovA when the bacteria were grown in the original TMH medium, but negatively controlled by PhoP when grown in the TMH containing 10μM MgCl_2_. Herein, an oligonucleotide primer, which was complementary to the RNA transcript of *rovA*, was employed to detect the primer extension product that represented the relative P1 promoter activity in the corresponding strains. The primer extension products were analyzed with 8 M urea−6% acrylamide sequencing gel. Lanes C, T, A, and G represent the Sanger sequencing reactions. Shown on the right side of the image is the transcription start site (nucleotide T, corresponding to the P1 promoter) that was located at 78 bp upstream of *rovA*. The P1 promoter was significantly repressed in *ΔrovA* relative to both *C-rovA* and WT gown in the original TMH; yet, it was significantly enhanced in *ΔphoP* relative to both *C-phoP* and WT grown in the TMH containing 10μM MgCl_2_ The P1 promoter was transcribed at almost the same level in every paired WT and complemented mutant. These results confirmed that the *phoP* or *rovA* mutation was nonpolar.
(TIF)
