# Supplementary figures and images for: Molecular Characterization of Transcriptional Regulation of rovA by PhoP and RovA in Yersinia pestis
Source: PLoS One. 2011 Sep 26;6(9):e25484. doi: 10.1371/journal.pone.0025484 (PMC3180457; doi:10.1371/journal.pone.0025484)

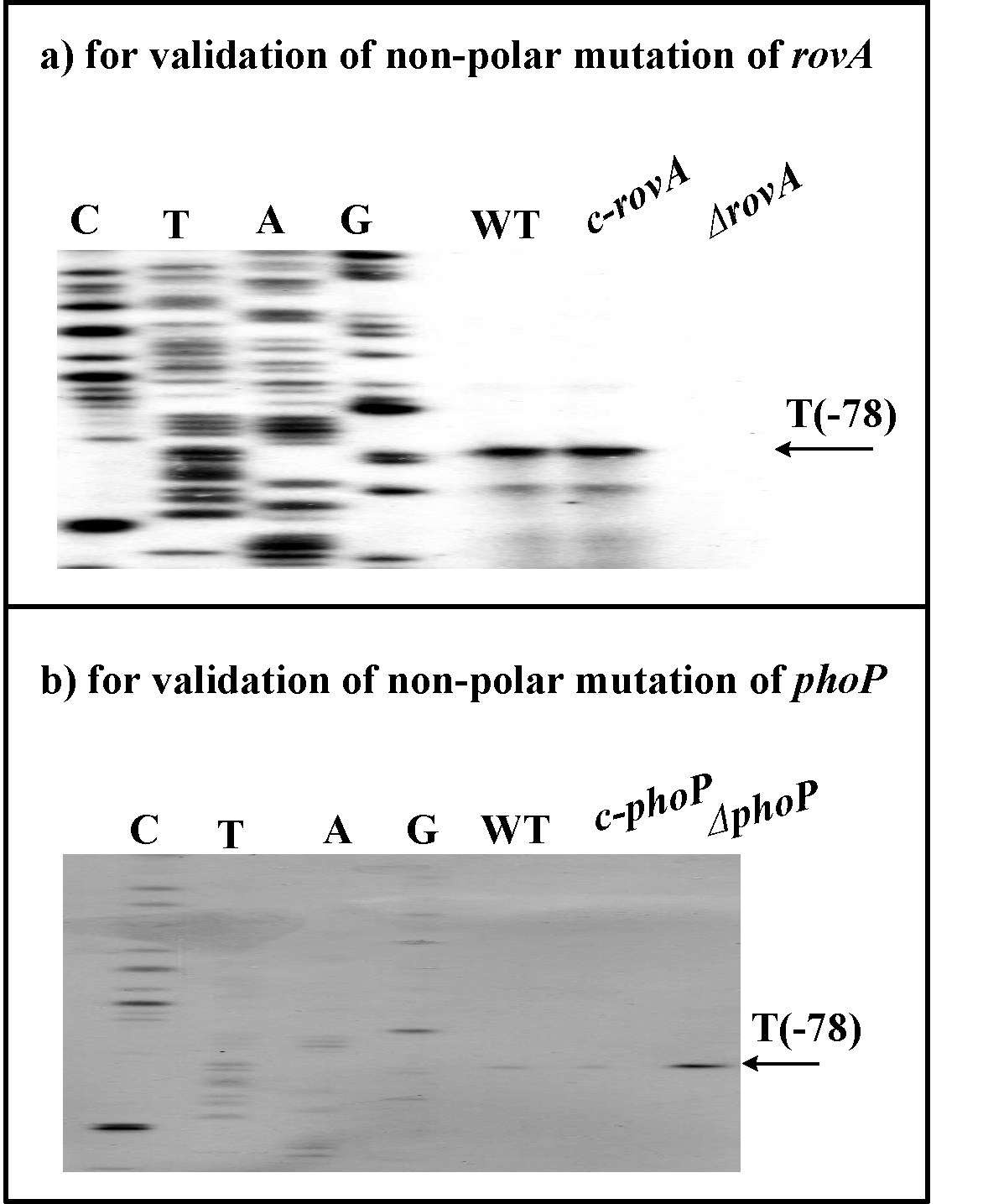

Supplement: Figure S1 — Primer extension assay for validation of non-polar mutation. The rovA or phop null mutant (ΔrovA or Δphop, respectively) was generated from the wild-type strain 201 (WT), and then the corresponding complemented mutant strain (C-rovA or C-phop, respectively) was constructed. As determined by several distinct methods (see the text of manuscript), the P1 promoter of rovA was positively regulated by RovA when the bacteria were grown in the original TMH medium, but negatively controlled by PhoP when grown in the TMH containing 10μM MgCl2. Herein, an oligonucleotide primer, which was complementary to the RNA transcript of rovA, was employed to detect the primer extension product that represented the relative P1 promoter activity in the corresponding strains. The primer extension products were analyzed with 8 M urea−6% acrylamide sequencing gel. Lanes C, T, A, and G represent the Sanger sequencing reactions. Shown on the right side of the image is the transcription start site (nucleotide T, corresponding to the P1 promoter) that was located at 78 bp upstream of rovA. The P1 promoter was significantly repressed in ΔrovA relative to both C-rovA and WT gown in the original TMH; yet, it was significantly enhanced in ΔphoP relative to both C-phoP and WT grown in the TMH containing 10μM MgCl2 The P1 promoter was transcribed at almost the same level in every paired WT and complemented mutant. These results confirmed that the phoP or rovA mutation was nonpolar. (TIF) [file pone.0025484.s001.tif]
